# Harm reduction in the Heartland: public knowledge and beliefs about naloxone in Nebraska, USA

**DOI:** 10.1186/s12954-022-00606-8

**Published:** 2022-03-04

**Authors:** Allison Schlosser, Patrick Habecker, Rick Bevins

**Affiliations:** 1 Sociology and Anthropology Department, University of Nebraska Omaha, Arts and Sciences Hall 383N, 6001 Dodge Street, Omaha, NE 68182 USA; 2 Rural Drug Addiction Research Center, University of Nebraska-Lincoln, Olfdather Hall – 4th Floor, 660 N 12th Street, Lincoln, NE 68588 USA; 3 Department of Psychology, University of Nebraska-Lincoln, 238 Burnett Hall, Lincoln, NE 68588-0308 USA

**Keywords:** Harm reduction, Naloxone, Narcan®, Opioid use, Rural health, United States

## Abstract

**Background:**

Opioid-related overdose deaths have been increasing in the United States (U.S.) in the last twenty years, creating a public health challenge. Take-home naloxone is an effective strategy for preventing opioid-related overdose death, but its widespread use is particularly challenging in smaller cities, towns, and rural areas where it may be stigmatized and/or poorly understood.

**Methods:**

We analyzed data on knowledge and beliefs about drug use and naloxone among the general public in Nebraska, a largely rural state in the Great Plains region of the U.S., drawing on the 2020 Nebraska Annual Social Indicators Survey.

**Results:**

Respondents reported negative beliefs about people who use drugs (PWUD) and little knowledge of naloxone. Over half reported that members of their community view PWUD as blameworthy, untrustworthy, and dangerous. Approximately 31% reported being unaware of naloxone. Only 15% reported knowing where to obtain naloxone and less than a quarter reported knowing how to use it. Knowing where to obtain naloxone is associated with access to opioids and knowing someone who has recently overdosed, but having ever used opioids or being close to someone who uses opioids is not associated with naloxone knowledge. Finally, almost a quarter of respondents endorsed the belief that people who use opioids will use more if they have access to naloxone.

**Conclusion:**

Our findings highlight stigmatizing beliefs about PWUD and underscore the need for further education on naloxone as an effective strategy to reduce opioid-related overdose death. We highlight the implications of these findings for public education efforts tailored to non-urban communities.

## Introduction

In the last twenty years, the United States (U.S.) has struggled to respond to what is now commonly referred to as an “overdose crisis.” In 2019, over 70,000 people died of a drug overdose in the U.S.—more than four times the 1999 total—and over two-thirds of these deaths involved an opioid [1]. Provisional data suggest an acceleration of overdose deaths during the novel coronavirus (COVID-19) pandemic. In the 12 months ending in April 2021, over 100,000 drug overdose deaths occurred; an increase of nearly 30% compared to the prior year [2].

Opioid-related overdose deaths can be prevented with the timely administration of naloxone, an opioid antagonist that quickly reverses opioid‐induced respiratory depression [3]. Take-home naloxone (THN)—the provision of naloxone to people who use opioids and others likely to witness an overdose and training on its administration—is a harm reduction intervention that aims to decrease the negative consequences of drug use without requiring its elimination [4] . In 2014, the World Health Organization recommended THN as a tool for the community management of opioid overdose [5]. The ongoing rise of opioid-related overdose deaths, however, highlights the insufficient adoption of THN as well as the need for greater investment in public awareness and support of this strategy to realize its full potential [5].

Notably, social stigma associated with drug use and “addiction” limits the accessibility of naloxone [6]. Despite a worsening overdose crisis and evidence of the public health benefits of naloxone, THN often faces significant political resistance, particularly in smaller cities, towns, and rural areas [7]. On this point, Ezell et al. [8] argue that stigma against people who use opioids and harm reduction interventions may be particularly problematic in rural areas because of decreased anonymity along with limited access and/or resistance to “neutralizing” information on these topics. This possibility underscores the need for more research on attitudes regarding opioid use and THN in rural areas that are increasingly affected by the overdose crisis [9].

Nebraska, a largely rural state in the Central Great Plains region of the U.S., has not been spared from the overdose crisis. In 2018, nearly 35% of the 138 overdose deaths in the state involved an opioid [10]. From October 2019 to October 2020, there was a 39.7% increase in all drug overdose deaths in the state, surpassing the 30% increase seen in the U.S. overall [11]. In 2015, a Nebraska law authorized prescribers and dispensers to distribute naloxone to family members, friends, and others who may be positioned to assist someone experiencing an opioid-related overdose and provided administrative, civil, and criminal immunity for individuals administering naloxone [12].

Despite these policy changes, THN has not been widely disseminated in the state [13]. To better understand this lack of adoption, this paper explores knowledge and beliefs about opioid use and naloxone among the general public in the state drawing on data from the 2020 Nebraska Annual Social Indicators Survey (NASIS).

## Methods

We use data from the NASIS, a paper survey mailed to an address-based sample of 8,000 adult Nebraskans (19 is the age of majority in Nebraska) in late July through November 2020. The 2020 NASIS sample was stratified by the six Behavioral Health Regions of the state plus Omaha and Lincoln, the two largest cities in the state. One thousand addresses were selected at random in each of the eight mutually exclusive strata. A map of the regions and a list of the ZIP codes used to define the borders of Omaha and Lincoln are available in the 2020 NASIS methodology report [14]. The University of Nebraska Lincoln Institutional Review Board approved this research (#20160816236FB).

The overall response rate for the 2020 NASIS was 27.7% (AAPOR RR2) with 11% of the mailings postmaster returned because of vacancies or other address ineligible codes. Due to the stratified sample design, weights are used to account for chance of selection by strata, within household probability of selection, nonresponse, and post-stratification for region, age, and gender. All analyses in this paper use weights in Stata 15 with the svy command. After removing cases with missing data, we have a sample of 1712. The average age of the sample was 49, ranging from 19 to 99, half of the sample were women, and 90% said they were White and not Hispanic. Fifty percent of the sample had a four-year degree or higher, 35% had some college or a technical degree, and 15% had a high school degree or less. Additionally, 53.4% percent of the sample reported household income greater than $75,000 in the past year, 19.8% reported $50,000-$75,000, and 26.8% reported less than $50,000.

## Results

We present results related to knowledge and beliefs about drug problems and access to opioids, people who use drugs (PWUD) and substance use disorder (SUD), and drug overdose and naloxone (see Table 1: Key survey questions and outcomes). We then present a multinomial logistic regression model predicting knowledge about where to get naloxone if a respondent needed it. We use the phrase “substance use disorder” in this report because this language was used in some NASIS survey questions. However, we acknowledge that this language may inaccurately communicate that the source of the disorder is in the individual and not the social and economic environment.

**Table 1:** Key survey questions and outcomes (n = 1712)

	Estimated percent	Estimate 95% confidence intervals	
How much of a problem do you think drug addiction is in the country today?			
Not at all a problem	0.5	0.2	1.5
A small problem	7.0	5.5	8.8
A moderately big problem	41.5	38.3	44.8
A very big problem	51.0	47.8	54.3
How much of a problem do you think drug addiction is in your local community today?			
Not at all a problem	3.0	2.1	4.4
A small problem	35.7	32.7	38.9
A moderately big problem	43.2	40.0	46.5
A very big problem	18.1	15.7	20.8
Have you ever used the following substances in ways other than through a prescription in your lifetime?			
Heroin	0.7	0.3	1.5
Opioids other than heroin	4.8	3.5	6.6
Combined	4.8	3.5	6.6
Could you get the following substances in ways other than through a prescription if you wanted to?			
Heroin	8.7	7.1	10.8
Opioids other than heroin	12.1	10.1	14.5
Combined	12.1	10.1	14.5
Do you know anyone close to you that currently uses any of these substances in ways that are not prescribed?			
Heroin	2.0	1.2	3.3
Opioids other than heroin	6.1	4.6	8.1
Combined	6.4	4.8	8.4
How much do you disagree or agree that a substance use disorder can be stopped at any time if the person truly wants to?			
Strongly disagree	22.3	19.7	25.0
Disagree	33.8	30.8	37.0
Neither disagree or agree	19.2	16.9	21.9
Agree	19.6	17.1	22.4
Strongly agree	5.1	3.8	6.8
Most people in my community believe that a person who uses cocaine, methamphetamine, opioids, or heroin cannot be trusted			
Strongly disagree	1.0	0.5	1.7
Disagree	3.2	2.3	4.4
Neither disagree or agree	16.4	14.1	18.9
Agree	54.7	51.4	57.9
Strongly agree	24.7	22.0	27.6
Most people in my community believe that a person who uses cocaine, methamphetamine, opioids, or heroin is dangerous			
Strongly disagree	0.9	0.5	1.6
Disagree	3.7	2.8	5.0
Neither disagree or agree	21.2	18.7	24.0
Agree	53.8	50.5	57.0
Strongly agree	20.4	17.9	23.1
Most people in my community believe that a person who uses cocaine, methamphetamine, opioids, or heroin is to blame for their own problems			
Strongly disagree	1.8	1.0	2.9
Disagree	7.9	6.3	9.7
Neither disagree or agree	26.6	23.9	29.5
Agree	49.3	46.1	52.6
Strongly agree	14.4	12.3	16.9
How much do you disagree or agree that you are able to recognize a person overdosing?			
Strongly disagree	4.9	3.7	6.6
Disagree	20.0	17.5	22.7
Neither disagree or agree	38.4	35.3	41.6
Agree	28.5	25.7	31.5
Strongly agree	8.2	6.5	10.3
Do you know anyone who has experienced a drug overdose in the past year?			
Yes	7.4	5.8	9.4
No	92.6	90.6	94.2
Do you know where to get Narcan® (naloxone) if you needed it?			
Yes	15.0	12.9	17.5
No	53.7	50.4	56.6
I don't know what this is	31.3	28.3	34.4

### Drug concerns and opioid access

Respondents expressed a high level of concern about drug use in the country and in their communities. Over 92% reported that drug use is a moderately big or very big problem in the U.S. overall. More than 61% also reported this level of concern about drug use in their local communities. Additionally, respondents reported the ability to access opioids in ways other than through a prescription: 8.7% reported the ability to obtain heroin and 12.1% reported the ability to obtain opioids other than heroin without a prescription. Furthermore, 2% reported knowing someone close to them that currently uses heroin and about 6% reported knowing someone close to them that currently uses opioids other than heroin. Only 0.7% of respondents reported ever using heroin in their lifetime, and 4.8% reported using opioids other than heroin in ways other than prescribed in their lifetime.

### Beliefs about SUD and PWUD

In addition to significant concern about drug use, respondents expressed potentially stigmatizing beliefs about SUD and PWUD. A quarter of the sample expressed the belief that having a SUD is a choice, agreeing or strongly agreeing that SUD can be stopped at any time if the person truly wants to quit (about 20% were neutral; 56% disagreed or strongly disagreed). Respondents also expressed negative attitudes about people who use cocaine, methamphetamines, and heroin or other opioids in their communities. The majority of respondents agreed or strongly agreed that most people in their community view these individuals as untrustworthy (79%), dangerous (74%), and deserving of blame (64%).

### Overdose and naloxone

Despite concern about the severity of drug problems in their local communities and knowledge of people who are currently using opioids, a quarter of respondents reported that they would be unable to recognize when a person is overdosing—almost 40% were neutral. Only 15% reported that they know where to get naloxone (also referred to as Narcan® on the survey) if they needed it. Further, less than a quarter (22%) reported that they know how to use naloxone. Moreover, approximately 31% reported being unaware of naloxone altogether. Finally, while just under a quarter of respondents reported the belief that people who use heroin or other opioids will use more of these drugs if they have access to naloxone, over half (58%) responded neutrally.

### Naloxone awareness: associations

To better understand patterns of naloxone knowledge, we used multinomial logistic regression to test associations between knowledge and several individual, geographic, and opioid-related factors (see Table 2: Multinomial regression model predicting knowledge of where to get naloxone). The model tests differences between people who know how to obtain naloxone (responded "Yes") and those who do not know what naloxone is, and the differences between those who do not know how to obtain naloxone (responded “No”) and those who do not know what naloxone is.

**Table 2:** Multinomial regression model predicting knowledge of where to get naloxone (n = 1712)

Measure	Model 1: "Yes" compared to "I don’t know what this [Narcan®] is"				Model 2: "No" compared to "I don’t know what this [Narcan®] is"			
	exp(b)	*p* value	95% CI		exp(b)	*p* value	95% CI	
Age	0.986	0.044	0.974	1.000	1.007	0.121	0.998	1.016
Political party								
Democrat	(reference)				(reference)			
Republican	0.657	0.117	0.388	1.111	0.654	0.033	0.442	0.967
Independent	0.674	0.192	0.372	1.220	0.876	0.551	0.566	1.354
Other party	0.869	0.799	0.294	2.569	0.796	0.571	0.362	1.751
Gender								
Male	(reference)				(reference)			
Female	1.904	0.003	1.247	2.907	1.166	0.322	0.860	1.581
Self-described race								
White and not Hispanic or Latino	1.6959	0.211	0.750	3.674	1.571	0.079	0.949	2.603
Not White	(reference)				(reference)			
Nebraska behavioral health regions with separate Omaha and Lincoln								
Panhandle	0.498	0.110	0.212	1.171	0.854	0.569	0.496	1.471
Southwest	0.994	0.988	0.434	2.272	0.938	0.952	0.559	1.729
South central	0.964	0.930	0.432	2.155	0.927	0.780	0.545	1.578
North	1.325	0.500	0.585	3.004	0.770	0.349	0.445	1.332
Southeast without Lincoln	1.222	0.599	0.578	2.580	0.797	0.424	0.456	1.391
Midlands without Omaha	0.722	0.394	0.341	1.528	0.807	0.395	0.493	1.323
Lincoln	1.000	0.999	0.493	2.025	0.912	0.722	0.548	1.516
Omaha	(reference)				(reference)			
Ever used opioids (Heroin/others)	0.949	0.935	0.270	3.338	1.631	0.299	0.647	4.111
Close to someone who uses opioids (Heroin/others)	1.045	0.922	0.432	2.526	1.021	0.959	0.465	2.243
Has access to opioids (Heroin/others)	3.608	0.000	1.890	6.886	1.328	0.312	0.767	2.299
Knows anyone who overdosed in past year	2.887	0.007	1.335	6.244	1.437	0.312	0.711	2.904
Intercept	0.419	0.147	0.129	1.360	1.028	0.946	0.459	2.304

We found that knowledge of naloxone is associated with age and gender. Older participants have lower odds of knowing how to get naloxone (*p* = 0.044) compared to not knowing what it is. Compared to men, women have 1.9 times greater odds of knowing where to get naloxone compared to not knowing what it is (*p* = 0.003). Additionally, knowing where to obtain naloxone is associated with access to opioids and knowing someone who has recently overdosed. Those who reported having access to opioids have 3.6 times higher odds of knowing where to get naloxone than reporting that they do not know what it is (*p* < 0.001). Respondents who know somebody who overdosed in the past year also have 2.9 times higher odds of knowing where to get naloxone than saying they do not know what it is (*p* = 0.007). However, having ever used opioids or being close to someone who uses opioids is not associated with naloxone knowledge. Nor were there differences by identifying as White or not, the region of Nebraska where the respondent lives, or if they live on a farm, open country, or in a town or city.

## Discussion

Our findings highlight the need for further education on PWUD, SUD, and naloxone as an effective harm reduction strategy to reduce opioid-related overdose death in Nebraska. We estimate that 6% of adults in the state know someone close to them who uses opioids without a prescription. This group represents a population for which knowledge of and access to naloxone can directly prevent overdose deaths. In regression testing, being close to someone who used opioids is not associated with naloxone knowledge, nor is a personal history of opioid misuse. However, having access to opioids and knowing somebody who overdosed in the past year is associated with higher odds of knowing where to find naloxone. The latter is encouraging as it suggests that people may learn about naloxone after an overdose in their personal social networks. However, a clear need for educational work in all areas of the state remains, as no single geographic measure we tested is associated with different levels of naloxone knowledge. This education could be facilitated by syringe service programs (SSPs): opportune settings through which to provide evidence-based, culturally relevant overdose education and naloxone distribution in addition to sterile syringes and other injection equipment [15]. SSPs, however, are not authorized in the state of Nebraska [16].

Additionally, over half of respondents reported that people in their community view individuals who use drugs as blameworthy, untrustworthy, and dangerous. This finding corroborates and extends research on stigmatizing beliefs about PWUD in rural areas [7, 8]. Ezell et al. [8] argue that stigma against PWUD may be intensified by residents’ limited interactions with these individuals and restricted information diffusion in rural communities (e.g., news deserts). They emphasize the need to improve public knowledge of the complex factors that contribute to opioid use as well as the moral and fiscal value of harm reduction in these areas.

Our findings highlight stigmatizing beliefs about PWUD and SUD, but also point to promising opportunities for public education. While almost a quarter of respondents expressed the belief that having a SUD is a choice, almost 20% were neutral and over half (56%) disagreed or strongly disagreed. Moreover, most respondents (58%) were neutral regarding the belief that naloxone access may cause increased drug use. These findings suggest that there may be some receptivity at this critical moment to educate the public about the experiences of PWUD, the complexities of SUD, and naloxone as a valuable tool for improving community health.

Efforts to destigmatize PWUD and SUD should be coupled with basic education on naloxone. Our findings indicate that most respondents are unable to identify when a person is overdosing, many are unaware of naloxone, and, of those who are aware of it, few know how to obtain or use THN. Specifically, our findings highlight the need for tailored education and outreach aimed at increasing naloxone knowledge among older individuals and men. Future efforts to promote THN in Nebraska could also benefit from the strategies that Childs and colleagues [7] suggest to promote harm reduction in non-urban communities: (1) identify local champions of harm reduction, (2) educate communities about harm reduction strategies before their implementation, (3) make harm reduction visible within these communities, and (4) secure “buy-in” from a variety of local stakeholders such as law enforcement and government officials.

Public awareness efforts should be informed by research on effective strategies to build public support for THN, such as combining harm reduction education with sympathetic portrayals of people who use opioids [17]. Naloxone may also be normalized using metaphors that frame it as similar to medications used to manage other chronic conditions (e.g., insulin for type 2 diabetes), medications used to respond to medical emergencies (e.g., epinephrine for allergic reactions), or other “just in case” tools (e.g., life insurance) [6]. These messages may be particularly effective in social media platforms to neutralize stigmatizing messages that are often circulated in these virtual social spaces.[8].

Additionally, research suggests that reducing opioid-related overdose may require a multi-strategy approach including improving Medication for Opioid Use Disorder (MOUD) access and retention in addition to naloxone distribution [18]. Yet, this approach is particularly challenging in states like Nebraska, where access to MOUD is limited and these medications often carry stigma [9]. Increasing access to and acceptability of MOUD, in addition to expanding naloxone awareness and distribution, will be essential to reducing opioid-related overdose in these communities.

## Limitations

While our findings provide valuable insight on public knowledge and beliefs about drug use and naloxone in Nebraska, the limitations of our mail survey methodology should be considered. The response rate of 27.7% means that survey results may not represent the state population. The sample was also 90% White and not Hispanic or Latino, higher than U.S. Census estimates of 78% for the state [19]. Finally, survey data are limited in its ability to capture nuances in knowledge and beliefs. Future qualitative research has potential to add depth and nuance to our findings.

## Conclusion

THN is now widely available in much of the U.S. The benefits of this strategy, however, may be limited by lack of knowledge and continued stigma of drug use. Our findings highlight these challenges, but also point to opportunities to address them through public education. Nebraska, like other largely rural U.S. states, has experienced a rise of opioid-related overdose during the COVID-19 pandemic. There is no better time to promote THN than now, as the public health impact of the pandemic has exacerbated the ongoing overdose crisis.

## Data Availability

The dataset supporting the conclusions of this article—the 2020 NASIS—will be publicly available by request to Bureau of Sociological Research after December 1, 2021 (https://bosr.unl.edu/nasis).

## Abbreviations

U.S.: United States
PWUD: People who use drugs
SUD: Substance use disorder
NASIS: Nebraska Annual Social Indicators Survey
THN: Take-home naloxone
MOUD: Medication for Opioid Use Disorder
